# Shuangqing Forum on carbon neutrality in construction and architecture

**DOI:** 10.1093/nsr/nwad110

**Published:** 2023-04-24

**Authors:** He Zhu

**Affiliations:** science and news editor at the editorial office of NSR, in Beijing, China

## Abstract

The Natural Science Foundation of China (NSFC) held the 326th Shuangqing Forum on 22 February 2023. As the first forum of 2023 and one of the first in-person activities post-COVID, a topic of national and global significance was chosen: the challenges, for basic sciences, of reaching carbon neutrality in construction and architecture. *National Science Review* (*NSR*) attended this forum, as several presentations related to materials science and information science would appeal to the broad audience of *NSR*. In the plenary session, four members of the Chinese Academy of Engineering (CAE), Profs. Jiaping Liu, Jianguo Wang, Weimin Zhuang and Hongyuan Mei, provided an overview of the challenges facing construction and architecture industries in the run-up to carbon neutrality.

## OVERVIEW

**Figure 1.**
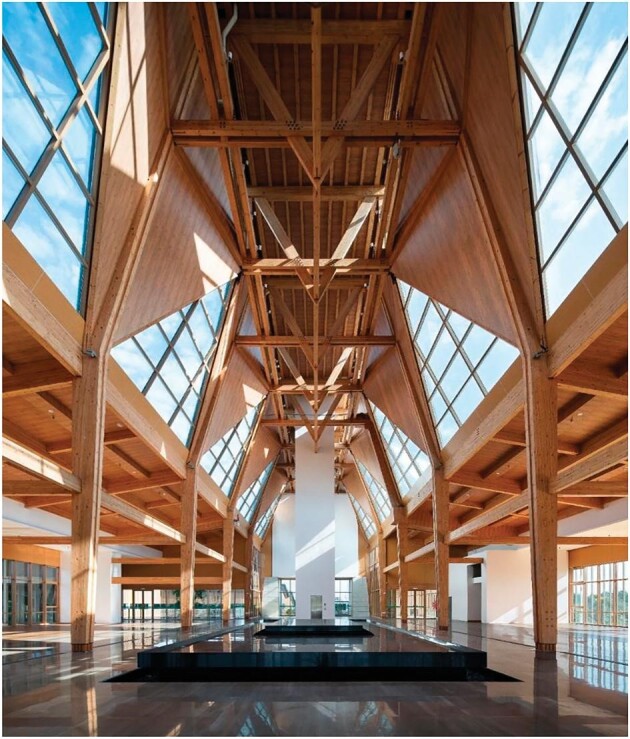
The main exhibition hall of the 10th Jiangsu Horticultural Exposition in Yangzhou, Jiangsu (*Courtesy of Wang Research Group*).

**Figure 2.**
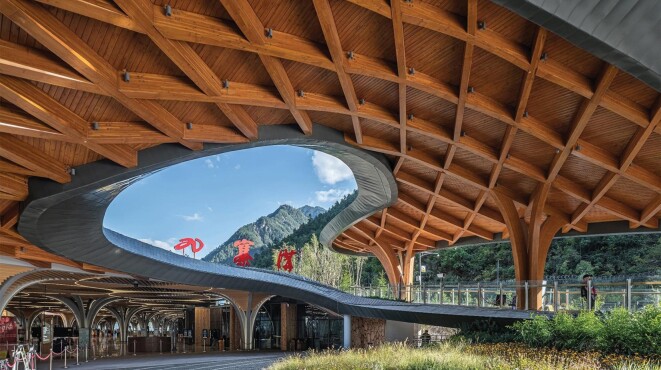
Jiuzhaigou Visitor Service Facilities, Sichuan (*Courtesy of Zhuang Research Group*).

China has made two major commitments in the effort to contain and combat climate change: reaching maximal carbon emissions (carbon peaking) in 2030 and reducing net carbon emissions to zero (carbon neutral) in 2060. These two goals have naturally created society-wide discussions on alternative power generation and storage, controlled nuclear fusion, the transition to electric transportation, the scaling potential of photovoltaic and electrolytic technologies, etc. One aspect that is receiving increasing attention is how these upcoming emission constraints affect construction and architecture industries as they are responsible for >50% of global carbon emissions. In response, construction engineers have established new policies such as a large-scale reduction of the use of steel and concrete, an increase in the use of steel and timber (Figs 1 and 2), the replacement of on-site building with assembly of pre-built parts, and more investment in novel environmentally friendly materials. On the other hand, architects have pointed out that during the economic expansion of recent decades, many architectural projects, including some ‘modern landmarks’, were not designed according to the principles of low emission. These ‘cautionary tales’ include extravagant office buildings constructed for small local governments and spectacular art or sports venues that unfortunately resulted in excessive maintenance costs. In addition, due to poor planning, some major public construction projects were demolished after only 20 to 30 years, leading to unnecessary reinvestments. Drawing lessons from these incidents, in order to reduce emissions, avoid waste and optimize operation during the lifetime of a construction project, modern analytical tools need to be used to strategize before construction and evaluate and monitor post-construction. One example of such modern low-emission planning is the quantitative comparison between demolishing an existing building and building a new one versus redesigning and repurposing it for a new application. The estimated energy saving is approximately a factor of 10, which favors a less exciting but more environmentally sensible choice.

Interestingly, one crucial challenge of meeting carbon peaking and carbon neutral goals is that they should not be based on experimental measurements but on numerical estimates. Considering that city-centric constructions and surrounding urban activities account for 75% of all emissions of greenhouse gases, accurate and country-wide estimates of carbon emissions will have to be initiated at the construction stage and scaled up from building to district, city and country. At the building stage, the comprehensive implementation of emission-saving technologies can not only improve the quality of residents’ lives, but also reduce emission of wastes and pollutants. Prof. Jiaping Liu of Xi’an University of Architecture and Technology (XAUAT) recommended we pay attention to the research on the basic science of green building design, starting with design principles and methods. However, the performance indexes of green buildings have not been included in the design code and mandatory provisions even though evaluation standards of green building have been developed and related work has been carried out in China for more than 20 years. This shortfall has greatly hindered the realization of the proposed emission goals. It is, therefore, imperative to implement ‘generalized building thermal design principles and methods’ to provide theoretical support for the systemic standards of the overall ‘greening’ of architectural designs.

In breakout sessions at the forum, experts from across China had detailed discussions on specific issues, such as how to compile an accurate database of the thermal parameters of construction materials, and methods of scaling emission estimates using artificial intelligence (AI).

## LOW-CARBON STRATEGIES BASED ON MATERIALS, DESIGN AND CONSERVATION

An accurate estimate of a building’s energy consumption requires precise modeling of heat transfer based on the materials’ thermal parameters such as thermal capacity and thermal conductivity. Prof. Yingying Wang of XAUAT discussed research trends and challenges related to construction materials. Carbon emissions produced by the normal usage of buildings account for 22% of emissions nationwide. There are currently three major methods of energy reduction: optimizing buildings themselves, updating appliances and incorporating renewable energy sources. Optimizing buildings themselves through thermal management receives the most attention from the engineering-physics research community. This step includes determining indoor and outdoor conditions and establishing design principles using a thermal conduction model. A central building block in this process is accurately determining the thermal parameters of construction materials. The team at XAUAT introduced a proper example of the basic science involved: a method to accurately estimate the thermal conductivity of building materials by modeling their moisture content. This was achieved using fractal theory, taking account of the porous and capillary structures of building materials.

Industrial buildings belong to a special category in the field of low emission construction and architecture as these designs must include uncommon elements such as sources of pollution, extreme heat and high-pressure airflow. Prof. Yi Wang at XAUAT provided an in-depth look at the unique challenges facing industrial constructions. Major topics in this field include how to balance safety with more complex designs of low emission, numerical simulations of industrial environments, recycling of exhaust heat, and highly efficient designs of ventilation systems. Specifically, improving ventilation design may result in a significant energy saving as ventilation accounts for over 50% of the energy consumption of industrial buildings. One scientific element here is non-invasive velocimetry, which safely and accurately maps airflow in three dimensions. Existing methods include using a doppler laser, particle tracking and schlieren imaging. Schlieren imaging is perhaps a more innovative method that is based on the dependency of the refractive index of air on its density. The experimental procedure consists of imaging a black and white calibration screen through a field of airflow and quantifying the change in light intensity. Using post-processing algorithms, velocity variations of turbulent airflow can be extracted from the intensity measurements.

In terms of reducing emissions for individual buildings, an ideal endpoint is sometimes referred to as PEDF, which stands for ‘photovoltaics, energy storage, direct current and flexibility’. Prof. Xiaohua Liu at Tsinghua University provided a description of the concept and an outlook for future directions. The photovoltaic step requires maximal usage of a building's surfaces for renewable energy acquisition, i.e. solar panels. These panels provide the foundation of the PEDF concept. The energy storage step consists of all possible resources as means of energy storage, including the batteries of the appliances inside the building and electric cars parked in the vicinity. The direct-current step emphasizes the usage of high-performance DC motors and DC/DC adaptors for other devices such as lights and computers. The final flexibility step refers to the transition of a building's role as an energy consumer to a composite and dynamic role of supplier-storage-consumer. Key challenges in PEDF include the large-scale transition from AC appliances to DC appliances and optimal allocation of battery capacities. The goal of PEDF is that it replaces the concept of ‘peak hours of electricity usage’ with a constant and ‘much reduced’ energy draw from the power grid, storing electricity drawn at night and spending it during the day. In addition, during peak hours of sunshine, it may send excess photovoltaic energy back into the power grid. Large-scale applications of PEDF will result in more efficient use of the power grid and reduced fuel consumption at power plants.

## SCALING METHODS AND COMPUTATIONAL DESIGNS

Producing emission estimates for a given building is a complex engineering problem. The sum can be divided into separate components, such as the manufacture of construction materials, transportation, construction, operation and maintenance. Prof. Qingqin Wang of the Chinese Academy of Building Research provided an overview of the existing methods of estimating emissions. They can be divided into two major categories: top-down methods and bottom-up methods. Top-down methods are initiated by values such as Gross Domestic Product (GDP), energy-related income and energy prices to produce a large-scale and macroscopic estimate. Then, using analytical downscaling methods, emission estimates of smaller units can be obtained. Top-down methods are better at reflecting macroeconomic factors but lack technical details. Bottom-up methods are initiated by calculating the emission value of an individual and representative building during a certain time period. By using upscaling methods, larger values of districts or cities can be obtained. The advantage of bottom-up methods is that they provide feedback on specific energy-saving policies so that policy makers can be better equipped to formulate energy-related regulations and strategies. Currently, the majority of countries adopt bottom-up methods.

Using emission estimates at various scales as inputs, an optimization method must be used to output preferred architectural designs. In the past, analytical methods were used to find the correlations between construction parameters and green performance in order to produce optimized strategies. However, with the increasing complexity of building parameters and requirements for the reduction of emissions, analytical solutions have become limited in their ability to support complex systems. Prof. Cheng Sun at the Harbin Institute of Technology introduced computational design methods to optimize green performance in construction and architecture. Similar computational approaches in physics, telecommunications and space exploration have proven to be successful, as advancements in computer hardware have merged with new methods in mathematics, engineering and computer science. Specific algorithms adopted in green architectural design include gradient descent, direct search, dynamic planning and genetic algorithms. In more recent years, AI has entered architectural design and made an impact in the same way it did in various other scientific disciplines. Specifically, researchers from around the world have implemented proxy modeling, parameterized modeling and predictive modeling using neural networks to optimize architectural designs. All these attempts have shown that AI-enabled design workflows can significantly simplify modeling procedures and reduce optimization time.

## OUTLOOK

Other topics discussed at this Shuangqing Forum included how to incorporate human experiences into quantitative architectural designs, how to properly include special urban features such as historical areas or high-density districts, and how to accommodate extreme weather conditions in architectural designs. As for concluding remarks, Prof. Jianguo Wang of Southeast University made one observation: since the onset of the industrial revolution, humanity has used chemical synthesis to produce construction materials such as steel, glass and cement. This marked the first cornerstone of construction and architecture, as these human-made materials greatly improved the efficiency of the building process, as well as the functions and quality of the final products. The second cornerstone will be the adoption of modern methods to produce the next generation of green construction materials to achieve the same goals with a much-reduced carbon footprint. The summary of Prof. Weimin Zhuang of Tsinghua University emphasized the importance of reducing emissions over full life cycles. Emission reduction will now become a precondition for construction industries, being initiated at the individual building level and scaling up in complexity to cities and beyond. In terms of achieving the most accurate estimates possible, data science and computational methods will be elevated from their current role as mere guidance to being the primary driving force. The overarching principle now is strategy and optimization before construction, and monitoring and evaluation post-construction.

## Notes

He Zhu (朱禾) is a science and news editor at the editorial office of NSR in Beijing, China

